# Unraveling the link between hypertension and depression in older adults: a meta-analysis

**DOI:** 10.3389/fpubh.2023.1302341

**Published:** 2023-11-24

**Authors:** Qingwen Gan, Ran Yu, Zerong Lian, Yiling Yuan, Yuanping Li, Lilan Zheng

**Affiliations:** ^1^School of Nursing, Nanchang University, Nanchang, China; ^2^Nursing Department, The First Affiliated Hospital of Nanchang University, Nanchang, China; ^3^Nursing Department, Heping Hospital Affiliated to Changzhi Medical College, Changzhi, China

**Keywords:** older adults, hypertension, depression, prevalence, risk factors, meta-analysis

## Abstract

**Objective:**

To perform a meta-analyses to understand the current status of and risk factors for depression in older adults with hypertension.

**Methods:**

Eight electronic databases and two clinical trial registries were searched to identify studies examining the incidence of and risk factors for depression among older adults with hypertension. The databases were searched from inception to June 2023. The included studies were evaluated using the Newcastle–Ottawa scale and the evaluation tool recommended by the Agency for Health care Research and Quality.

**Results:**

A total of 18 studies with 29,694 patients were included. Meta-analysis results showed that the prevalence of depression in older adults with hypertension was 29%. The risk factors for depression among this population included sex [OR value 95% confidence interval 2.24 (1.32, 3.82)], education level [OR 95% CI 1.79 (1.02, 3.14)], residence [OR 95% CI 1.37 (1.24, 1.52)], comorbidities [OR 95% CI 1.79 (1.69, 1.90)], hypertension classification [OR 95% CI 2.81 (1.79, 4.42)], marital status [OR 95% CI 1.50 (1.33, 1.69)], sleep status [OR 95% CI 2.86 (2.21, 3.69)], activity limitation [OR 95% CI 3.42 (2.84, 4.13)], drinking [OR 95% CI 2.25 (1.58, 3.19)], social support [OR 95% CI 3.26 (2.42, 4.41)], living alone [OR 95% CI 1.79 (1.57, 2.04)], stressful events [OR 95% CI 1.62 (1.39, 1.90)], and course of diseases [OR 95% CI 3.23 (2.10, 4.97)].

**Conclusion:**

The incidence of depression in older adults with hypertension is high, and there are many risk factors. Clinical health care professionals should intervene early to target the above risk factors to reduce the incidence of depression in older adults with hypertension worldwide.

**Systematic review registration:**

PROSPERO (york.ac.uk), identifier [CRD42023417106].

## 1 Introduction

With the decline in the global fertility rate and the increase in human life expectancy, the global population of older adults is increasing; some studies have estimated that the global population of older adults will reach 2.1 billion by 2050, which is twice the global population of older individuals in 2017 (1, 2). Older adults have a high prevalence of chronic diseases; in particular, the prevalence of hypertension can be as high as 64.23% (3). Some studies have shown that the prevalence of hypertension increases with age (4). The reason for this relationship may be that as adults age and experience changes in body function, they suffer from vascular sclerosis, decreased vascular elasticity, and reduced vascular ability to regulate and buffer blood pressure, thus causing hypertension (5). In addition, You et al. (6) showed that in the process of aging, the muscle structure and function of older people undergo significant changes, such as a decline in the level of muscle mass; furthermore, the decline in the level of muscle mass has an effect on the quality of sleep, and sleep disorders and hypertension are closely linked (7).

When older adult people experience chronic hypertensive diseases, they are prone to high levels of psychological stress and negative emotions, with depression being the most common (8). Li et al. showed (9) that with increasing age and blood pressure, neuronal loss in the CA1 region and elevated expression of the inflammatory factors COX-2 and MCP-1 were evident in hypertensive rats; furthermore, elevated COX-2 can lead to hippocampal neuronal damage, which is the main cause of depression (10). Choi et al. showed that TNF-α levels are elevated in hypertensive patients, and TNF-α can be secreted by glial cells and neurons, which has an important role in regulating neurotransmitters and controlling synaptic transmission, and abnormal TNF-α levels affect the mental symptoms of individuals and increase the risk of depression in individuals (11). Studies have also shown that elevated COX-2, an inflammatory mediator in hypertensive patients, affects the activity of the inflammatory factor IFN-γ. The IFN-γ factor can reduce the availability of BH4, which is a key enzyme cofactor for monoamine transmitter synthesis, and can limit the synthesis of the monoamines 5-HT, NE and DA; furthermore, abnormal levels of the monoamines 5-HT, NE and DA are strongly associated with the development of depression (12). In addition, sedentary behavior and irregular physical activity are common among older adults with hypertension, and studies have confirmed that prolonged sedentary behavior and irregular physical activity not only accelerate the process of individual aging (13) but also affect cognitive function and mental health (14, 15), thereby increasing the risk of depression in older adults with hypertension.

When depression occurs in older adults with hypertension, it not only increases the risk of suicide (16) but also has adverse effects on the immune system, digestive system, cardiovascular system, and endocrine system of patients, such as the presence of a reduced number of immune cells and low immune function; gastrointestinal disorders and loss of appetite; dyslipidemia and heart rate disorders; and abnormal secretion of catecholamine neurotransmitters and endocrine system disorders, all of which increase the difficulty of hypertension control in older adults and seriously affect the treatment and prognosis of older adults with hypertension (17, 18). Therefore, improving the quality of life of older adults with hypertension and reducing the incidence of depression in older adults with hypertension are prominent global public health issues.

At present, the incidence of depression in older adults with hypertension has been inconsistently reported, and studies on risk factors have yielded inconsistent results, thus making it impossible to provide an accurate and valid basis for health care professionals to screen older adults with hypertension who are at high risk of depression. Meta-analysis is the process of quantitatively synthesizing the results of multiple similar studies. Compared with the traditional review, it increases the sample content, improves the efficiency of statistical testing, and evaluates the consistency of the results of multiple studies, thus making its findings more accurate and reliable. Therefore, this study comprehensively integrates studies on the current status and risk factors for depression in older adults with hypertension through meta-analysis to provide evidence-based guidance for the early screening, prevention, and management of depression in older adults with hypertension around the world.

## 2 Methods

### 2.1 Study profile

The study was produced and reported in strict accordance with Preferred Reporting Items for Systematic Reviews and Meta-Analyses (PRISMA) statements (19) and was registered on the PROSPERO platform under the registration number CRD42023417106.

### 2.2 Literature search

Two researchers independently searched the China National Knowledge Infrastructure (CNKI), Wanfang Database, VIP Database, Sinomed, PubMed, Web of Science, Cochrane Library, and Embase databases to identify relevant studies on the incidence of and risk factors for depression in older adults with hypertension. The researchers also searched two clinical trial registries (Clinical Trials. gov and Chinese Clinical Trail Registry) to identify relevant unpublished studies. The databases were searched from inception to June 2023. Two investigators screened the studies based on the study designs, and the final search strategy was determined after a preliminary search and discussion among study members, with three core search terms: (1) hypertension (e.g., high blood pressure, high blood pressures, etc.); (2) depression (e.g., depressed, etc.); and (3) risk factors (e.g., related factor^*^, influence factor^*^, factor^*^, etc.). The specific search strategies can be found in Supplementary material 1.

### 2.3 Inclusion and exclusion criteria

The inclusion criteria were as follows: (a) study population: patients diagnosed with hypertension by diagnostic criteria (exclusion of a history of depression), mean age ≥ 60 years; (b) study content: studies involving the incidence of and risk factors for depression among older adults with hypertension; (c) outcome indicators: the incidence of depression and/or risk factors for depression in older adults with hypertension, with odds ratio values and 95% confidence intervals or raw data that can be converted for risk factors; and (d) study type: cross-sectional studies, case–controlled studies, or cohort studies.

The exclusion criteria were as follows: (a) duplicate literature; (b) reviews, meta-analysis, and graduation thesis; (c) literature with missing valid data; and (d) literature with low quality.

### 2.4 Literature screening and extraction

The retrieved studies were imported into EndNote literature management software by 2 systematically trained investigators (Qingwen Gan and Zerong Lian), who used the software and manually removed duplicates. Two investigators (Qingwen Gan and Ran Yu) carefully read the abstracts and full texts of the studies to select eligible articles. Two investigators (Ran Yu and Zerong Lain) extracted basic information about the included literature, including study, study type, country, sample size, incidence of depression, assessment tool, risk factors, etc. The entire process of literature screening and extraction was performed independently by 2 individuals, and a third party (Lilan Zheng) was consulted in cases of disagreement.

### 2.5 Quality evaluation of literature

The quality of the included case–control studies and cohort studies was evaluated by 2 investigators using the Newcastle–Ottawa scale (NOS) (20). The maximum score of the NOS is 9, and studies with a score of <5 are considered low-quality studies. The evaluation tool recommended by the American Agency for Health care Quality and Research (AHRQ) was used to evaluate the quality of the included cross-sectional studies (21). The maximum score of the AHRQ is 11, and studies with a score <6 are considered low-quality studies. Low-quality studies were excluded from this study. Three investigators rated the quality of evidence in this study using the Grading of Recommendations, Assessment, Development, and Evaluation (GRADE) system (22).

### 2.6 Statistical analysis

Reviewer Manager 5.4 and Stata 17.0 software were used to analyze the data. The heterogeneity of the included studies was tested by the Q test and I^2^. When *P* > 0.1 and I^2^ ≤ 50% heterogeneity was small, a fixed effects model was used for combined analysis; when *P* < 0.1 or I^2^ > 50% statistical heterogeneity existed, sensitivity analysis was applied to find the source of heterogeneity or subgroup analysis was performed to reduce study heterogeneity. If statistical heterogeneity still existed, a random effects model was selected for statistical analysis. Publication bias was analyzed by drawing funnel plots and performing Egger's test, and differences were considered statistically significant at *P* < 0.05.

## 3 Results

### 3.1 Search results

**Figure 1:**
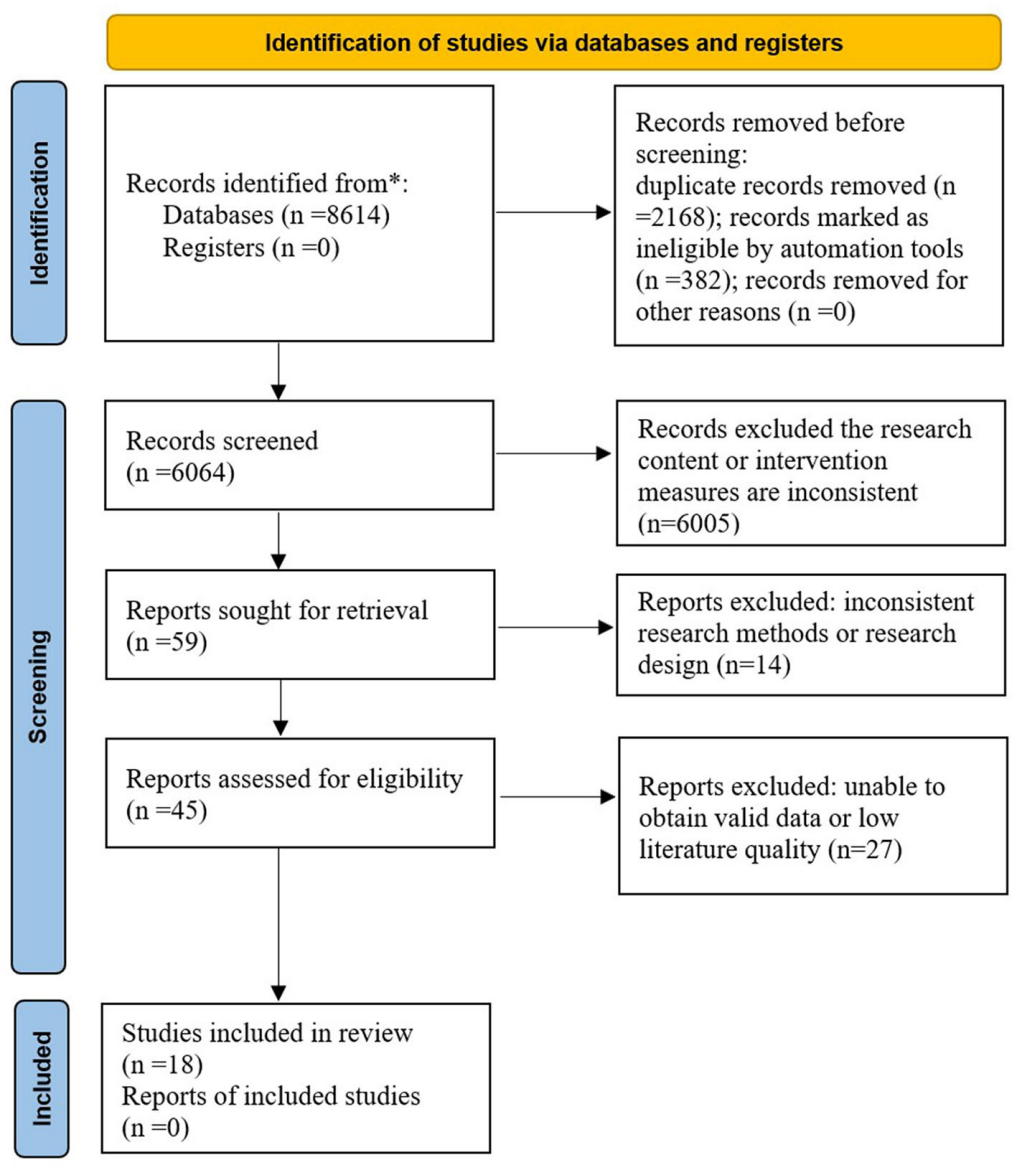
Flow chart of literature screening.

The search initially yielded 8,614 studies. A total of 2,168 duplicate studies, 382 review studies, and 6,005 studies that did not match the research content were excluded. After reading the full texts of the remaining 59 studies, 41 studies were excluded due to low quality, a lack of valid data, and inconsistent research designs. Ultimately, 18 studies were included (23–40). The specific screening process for the literature is shown in Figure 1.

### 3.2 Basic characteristics of included literature

A total of 18 studies were included (23–40), including 2 case–control studies (23, 27), 1 cohort study (26), and 15 cross-sectional studies (24, 25, 28–40), with a total of 29,694 patients and 24 risk factors. The highest score for quality assessment of the literature was 9 (38), and the lowest score was 6 (25, 27). The basic characteristics of the included studies are shown in Table 1.

**Table 1:** Basic characteristics of the included studies.

**References**	**Study type**	**Country**	**Total size (*n*)**	**Incidence rate (%)**	**Assessment tool**	**Risk factors**	**Quality evaluation**
Liu et al. (23)	Case–control	China	265	30.94	HAMD-17	1,2,4,5,13,20	7
Su et al. (24)	Cross-sectional	China	3,490	23.98	CES-D	3,4,6,7,8,9,10,18, 19	8
Ding et al. (25)	Cross-sectional	China	273	20.88	PHQ-9	1,2,6,7,11,16,20	6
Yang et al. (26)	Cohort study	China	3,386	35.65	CES-D 10	1,2,3,10,14,19	7
Ma et al. (27)	Case–control	China	100	42.00	HAMD-24	8	6
Du et al. (28)	Cross-sectional	China	200	61.00	SDS	5,6,7,9,15,18	7
Lei et al. (29)	Cross-sectional	China	846	37.71	GDS	4,12	8
Jin et al. (30)	Cross-sectional	China	182	47.80	HAMD-24	13,15,17	7
Bai and Jiang (31)	Cross-sectional	China	106	27.36	PHQ-9	4,5,9,17	7
Ling (32)	Cross-sectional	China	771	32.04	PHQ-9	1,2,6,11,13,15	8
Zheng et al. (33)	Cross-sectional	China	147	44.22	HRSD	2,7,12	7
Rosas et al. (34)	Cross-sectional	Brazil	438	22.37	GDS	7,14	8
Ma (35)	Cross-sectional	China	942	40.55	CES-D	1,4,6,11,12,13,16, 24	8
Agustini et al. (36)	Cross-sectional	Australia America	14,195	9.96	CES-D 10	22	7
Son et al. (37)	Cross-sectional	Korea	846	10.05	PHQ-9	2,7,8	7
Boima et al. (38)	Cross-sectional	Ghana	1,239	8.39	Clinical diagnosis	6,10,11,23	9
Gray et al. (39)	Cross-sectional	America	1,204	35.38	GDS	4,10,11,21	8
Ma et al. (40)	Cross-sectional	China	1,064	14.94	CES-D	2,3,6,7,8,11,14	8

HAMD, Hamilton Depression Scale; CES-D, Center for Epidemiologic Studies-Depression; PHQ-9, Patient Health Questionnaire-9; SDS, Self-rating Depression Scale; GDS, Geriatric Depression Scale; HRSD, Hamilton Depression Rating Scale for Depression.

1, sex; 2, education level; 3, residence; 4, comorbidities; 5, hypertension classification; 6, marital status; 7, sleep status; 8, activity limitation; 9, drink; 10, self-rated health; 11, monthly income; 12, social support; 13,living alone; 14, stressful events; 15, course of diseases; 16, clinical symptoms; 17, total serum protein; 18, smoking; 19, medical insurance; 20, treatment adherence; 21, race; 22, beta-blockers; 23, religion; 24, independence.

### 3.3 Literature quality evaluation

The quality scores of all 18 studies included were ≥6 points. Except for two studies with a quality score of 6 points (25, 27), all other studies had a quality score of ≥7 points. The overall quality of the literature was relatively high, as shown in Table 1.

### 3.4 Meta-analysis of the incidence of depression in older adults with hypertension

#### 3.4.1 Incidence rate

**Figure 2:**
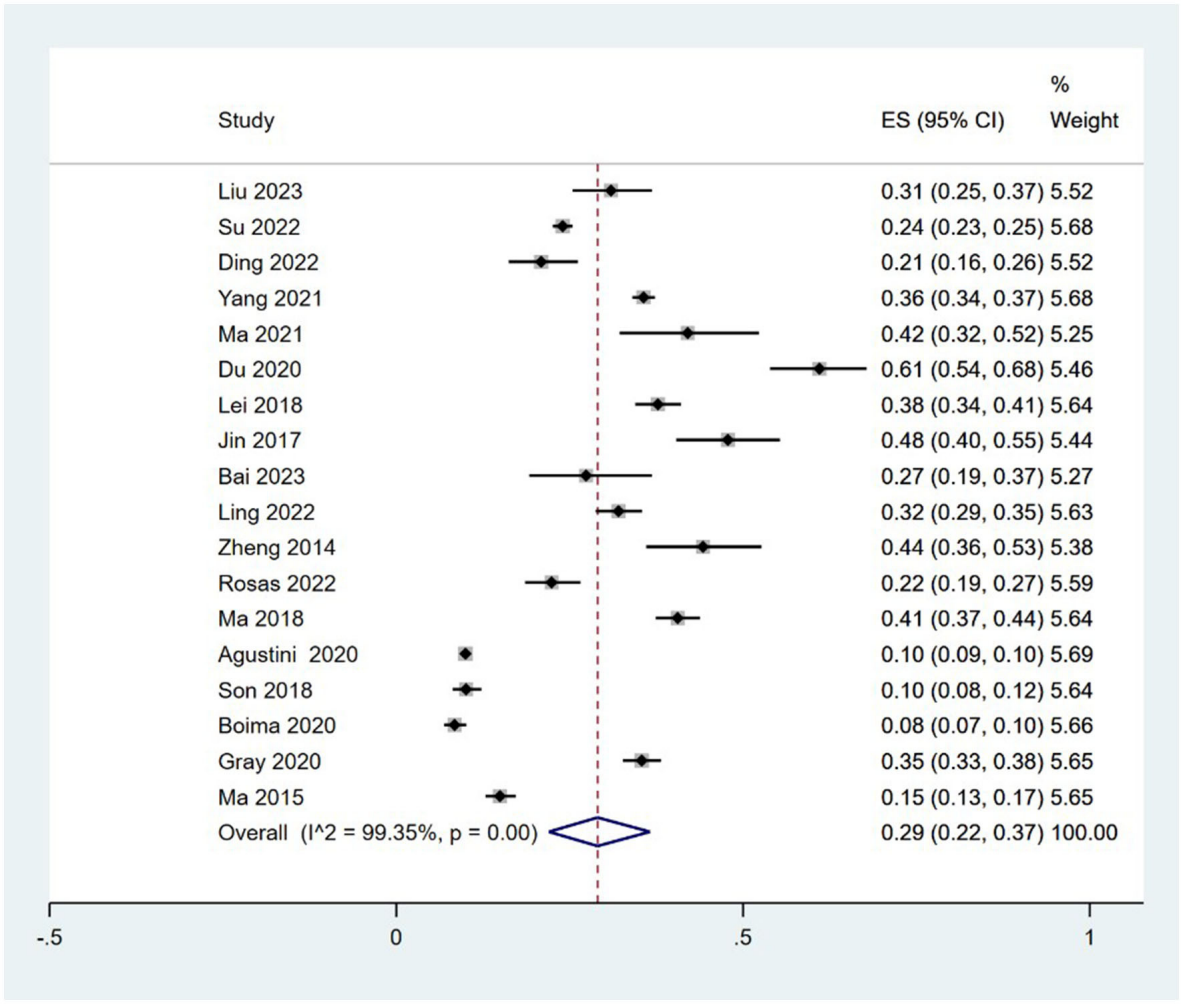
Forest plot of the incidence of depression in older adults with hypertension.

A total of 18 studies (23–40) analyzed the incidence of depression in older adults with hypertension (8.39–61.00%), and there was statistical heterogeneity among studies (I^2^ = 99.35%, *P* = 0.00); therefore, a random effects model was selected for the combined analysis. Meta-analysis revealed that the incidence of depression in older adults with hypertension was 29% [95% CI (22, 37%)], and the difference was statistically significant (*P* < 0.001). These findings are shown in Figure 2.

#### 3.4.2 Subgroup analysis

Subgroup analysis is carried out by country, study type, and assessment tool. The meta-analysis revealed that the incidence of depression in older adults with hypertension in China is 35% [95% CI (29%, 41%)], and the incidence of depression in older adults with hypertension in other regions is 16% [95% CI (8%, 26%)]. In the cross-sectional study of older adults with hypertension, the incidence of depression is 28% [95% CI (20%, 36%)], and the incidence of depression in older adults with hypertension in the case–control study is 34% [95% CI (29%, 39%)]. Regarding the HAMD-24 scale, which is used to evaluate depression in older adults with hypertension, the incidence rate is 46% [95% CI (40%, 52%)]; for the CES-D scale, the incidence of depression was 26% [95% CI (15%, 39%)]; for the CES-D10 scale, the incidence of depression was 14% [95% CI (13%, 14%)]; for the PHQ-9 scale, the incidence of depression was 22% [95% CI (10%, 36%)]; and for the GDS scale, the incidence of depression was 32% [95% CI (24%, 40%)]. The specific subgroup analysis results are shown in Table 2.

**Table 2:** Subgroup analysis results of depression incidence in older adults with hypertension.

**Subgroup analysis**	**Studies (*n*)**	**Heterogeneity test**		**Effect-model**	**Meta-analysis result**	
		**I** ^2^ **/%**	***P*** **-value**		**OR (95% CI)**	***P*** **-value**
**Country**						
China	13	97.39	<0.001	Random effects	0.35 (0.29, 0.41)	<0.001
Other countries	5	99.19	<0.001	Random effects	0.16 (0.08, 0.26)	<0.001
**Study type**						
Cross-sectional study	15	99.27	<0.001	Random effects	0.28 (0.20, 0.36)	<0.001
Case–control Study	2	0	<0.001	Fixed effects	0.34 (0.29, 0.39)	<0.001
**Assessment tool**						
HAMD-24	2	0	<0.001	Fixed effects	0.46 (0.40, 0.52)	<0.001
CES-D	3	98.85	<0.001	Random effects	0.26 (0.15, 0.39)	<0.001
CES-D 10	2	0	<0.001	Fixed effects	0.14 (0.13, 0.14)	<0.001
PHQ-9	4	97.68	<0.001	Random effects	0.22 (0.10, 0.36)	<0.001
GDS	3	94.33	<0.001	Random effects	0.32 (0.24, 0.40)	<0.001

HAMD-24, Hamilton Depression Scale-24; CES-D, Center for Epidemiologic Studies-Depression; CES-D 10, Center for Epidemiologic Studies-Depression 10; PHQ-9, Patient Health Questionnaire-9; GDS, Geriatric Depression Scale; OR, odds ratios; CI, confidence intervals.

### 3.5 Meta-analysis of risk factors for depression in older adults with hypertension

There were 24 risk factors in this study, of which clinical symptoms, total serum protein, smoking, medical insurance, treatment adherence, race, beta-blockers, religion, and independence were not meta-analyzed because of the limited number of studies included in each. The results of the study showed that female sex, low education level, rural areas, comorbidities, high hypertension classification, non-marital status, poor sleep quality, activity limitation, drinking, low social support, living alone, stressful events, and a long course of disease were risk factors for the occurrence of depression in older adults with hypertension. Additionally, the correlations of self-rated health and monthly income with the occurrence of depression in older adults with hypertension were not significant. The specific analysis results and quality of evidence ratings are shown in Table 3.

**Table 3:** Meta-analysis of risk factors for depression in older adults with hypertension.

**Influence factor**	**Studies (*n*)**	**Heterogeneity test**		**Effect-model**	**Meta-analysis result**		**Level of evidence**
		***P*** **-value**	**I** ^2^ **/%**		**OR (95% CI)**	***P*** **-value**	
Sex	5	0.04	64	Random effects	2.24 (1.32, 3.82)	0.003	Low
Education level	7	0.01	67	Random effects	1.79 (1.02, 3.14)	0.04	Low
Residence	3	0.52	0	Fixed effects	1.37 (1.24, 1.52)	<0.001	Low
Comorbidities	6	0.01	68	Random effects	1.79 (1.69, 1.90)	<0.001	Low
Hypertension classification	3	0.93	0	Fixed effects	2.81 (1.79, 4.42)	<0.001	Low
Marital status	7	0.11	42	Fixed effects	1.50 (1.33, 1.69)	<0.001	Low
Sleep status	7	0.23	27	Fixed effects	2.86 (2.21, 3.69)	<0.001	Low
Activity limitation	4	0.54	0	Fixed effects	3.42 (2.84, 4.13)	<0.001	Low
Drink	3	0.14	55	Random effects	2.25 (1.58, 3.19)	<0.001	Low
Self-rated health	4	<0.001	93	Random effects	0.88 (0.58, 1.34)	0.56	Very low
Monthly income	6	<0.001	86	Random effects	1.20 (0.76, 1.90)	0.44	Very low
Social support	3	0.07	62	Random effects	3.26 (2.42, 4.41)	<0.001	Low
Living alone	4	0.50	0	Fixed effects	1.79 (1.57, 2.04)	<0.001	Low
Stressful events	3	0.60	0	Fixed effects	1.62 (1.39, 1.90)	<0.001	Low
Course of disease	3	0.54	0	Fixed effects	3.23 (2.10, 4.97)	<0.001	Moderate

OR, odds ratios; CI, confidence intervals.

#### 3.5.1 Social demography factors

**Figure 3:**
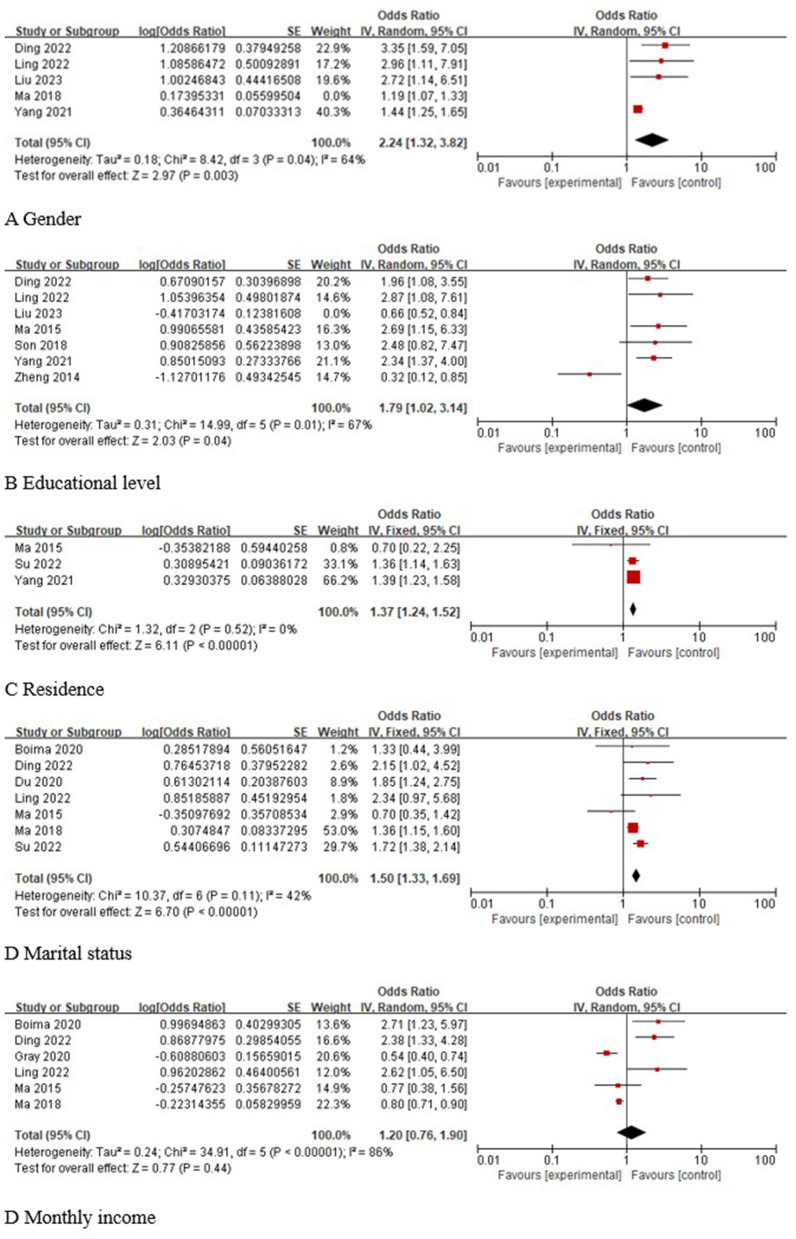
Meta-analysis of social demography factors.

A total of five studies (23, 25, 26, 32, 35) reported the association of sex with the occurrence of depression in older adults with hypertension, and statistical heterogeneity was found across studies (I^2^ = 75%, *P* = 0.003). Sensitivity analysis was performed and revealed that the study by Ma 2018 (24) was a source of heterogeneity. After excluding this study and reperforming the pooled analyses, the results showed that female sex was a risk factor for depression in older adults with hypertension [OR 95% CI 2.24 (1.32, 3.82), *P* = 0.003]. A total of seven studies (23, 25, 26, 32, 33, 37, 40) reported the correlation between education level and the occurrence of depression in older adults with hypertension, and statistical heterogeneity was found across studies (I^2^ = 86%, *P* < 0.001). Sensitivity analysis was performed and revealed that the study by Liu 2023 (23) was a source of heterogeneity. After excluding this study and reperforming the pooled analyses, the results showed that a low level of education was a risk factor for depression in older adults with hypertension [OR 95% CI 1.79 (1.02, 3.14), *P* = 0.04]. A total of three studies (24, 26, 40) reported the association between place of residence and the occurrence of depression in older adults with hypertension, and the results of the meta-analysis showed that rural area was a risk factor for depression in older adults with hypertension [OR 95% CI 1.37 (1.24, 1.52), *P* < 0.001]. A total of seven studies (24, 25, 28, 32, 35, 38, 40) analyzed the correlation between marital status and the occurrence of depression in older adults with hypertension, and the results of the meta-analysis showed that being unmarried was a risk factor for depression in older adults with hypertension [OR 95% CI 1.50 (1.33, 1.69), *P* < 0.001]. A total of six studies (25, 32, 35, 38–40) analyzed the correlation between monthly income and the occurrence of depression in older adults with hypertension, and the results of the meta-analysis showed that the correlation between monthly income and the occurrence of depression in older adults with hypertension was not significant [OR 95% CI 1.20 (0.76, 1.90), *P* = 0.44]. The results of the specific analysis are shown in Figure 3.

#### 3.5.2 Disease factors

**Figure 4:**
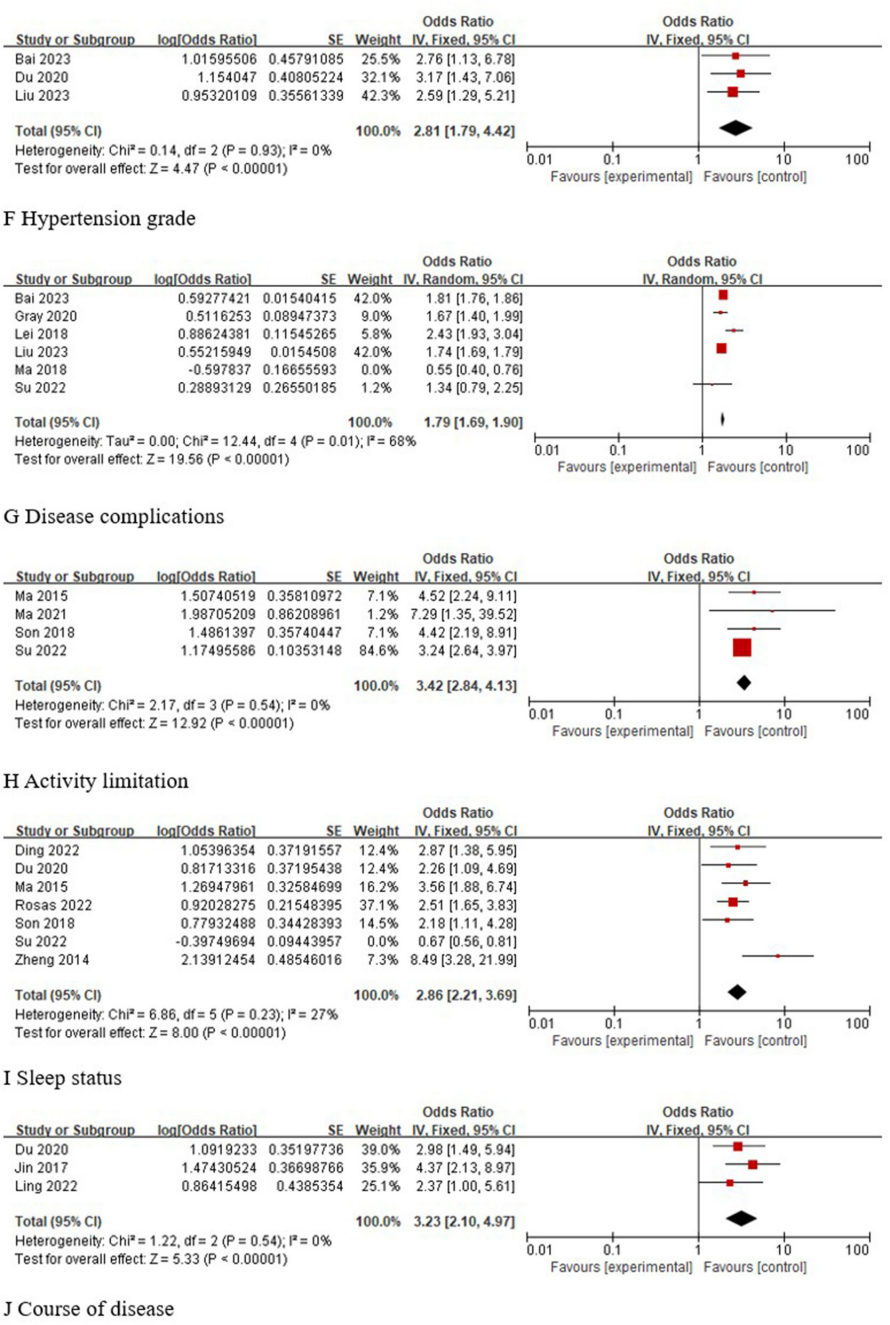
Meta-analysis of disease factors.

A total of three studies (23, 28, 31) analyzed the correlation between hypertension classification and the occurrence of depression in older adults with hypertension, and the results of the meta-analysis showed that high hypertension classification was a risk factor for depression in older adults with hypertension [OR 95% CI 2.81 (1.79, 4.42), *P* < 0.001]. A total of six studies (23, 24, 29, 31, 35, 39) analyzed the correlation between comorbidities and the occurrence of depression in older adults with hypertension, and statistical heterogeneity (I^2^ = 92%, *P* < 0.001) was found across studies. Sensitivity analysis was performed and revealed that the study by Ma 2018 (35) was a source of heterogeneity. After excluding this study and performing the pooled analyses, the results showed that comorbidities were a risk factor for depression in older adults with hypertension [OR 95% CI 1.79 (1.69, 1.90), *P* < 0.001]. A total of four studies (24, 27, 37, 40) analyzed the correlation between activity limitation and the occurrence of depression in older adults with hypertension, and the results of the meta-analysis showed that activity limitation was a risk factor for depression in older adults with hypertension [OR 95% CI 3.42 (2.84, 4.13), *P* < 0.001]. A total of seven studies (24, 25, 28, 33, 34, 37, 40) analyzed the correlation between sleep status and the occurrence of depression in older adults with hypertension, and statistical heterogeneity was found across studies (I^2^ = 93%, *P* < 0.001). Sensitivity analysis was performed and revealed that the study by Su 2022 (24) was a source of heterogeneity. After excluding this study, the pooled analyses were performed again, and the results showed that poor sleep quality was a risk factor for depression in older adults with hypertension [OR 95% CI 2.86 (2.21, 3.69), *P* < 0.001]. A total of three studies (28, 30, 32) analyzed the correlation between the course of disease and the occurrence of depression in older adults with hypertension, and the results of the meta-analysis showed that a long course of disease was a risk factor for depression in older adults with hypertension [OR 95% CI 3.23 (2.10, 4.97), *P* < 0.001]. These findings are shown in Figure 4.

#### 3.5.3 Other factors

**Figure 5:**
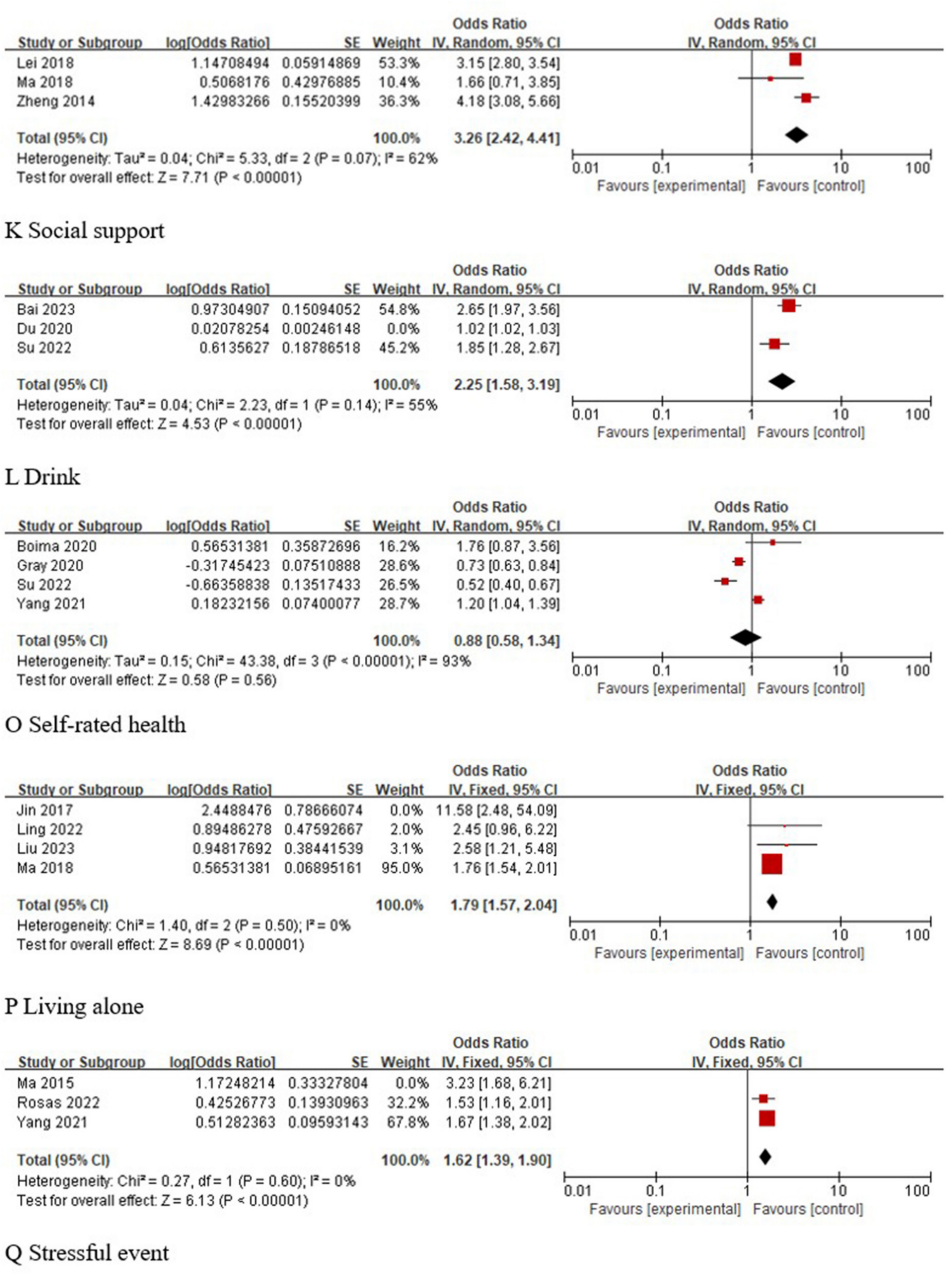
Meta-analysis of other factors.

A total of three studies (29, 33, 35) analyzed the correlation between social support and the occurrence of depression in older adults with hypertension, and the results of the meta-analysis showed that low social support was a risk factor for depression in older adults with hypertension [OR 95% CI 3.26 (2.42, 4.41), *P* < 0.001]. A total of three studies (24, 28, 31) analyzed the association between alcohol consumption and the occurrence of depression in older adults with hypertension, and statistical heterogeneity was found across studies (I^2^ = 96%, *P* < 0.001). Sensitivity analysis was performed and revealed that the study by Du 2020 (28) was a source of heterogeneity. After excluding this study, the pooled analyses were performed again, and the results showed that drinking was a risk factor for depression in older adults with hypertension [OR 95% CI 2.25 (1.58, 3.19), *P* < 0.001]. A total of four studies (24, 26, 38, 39) analyzed the correlation between self-rated health and the occurrence of depression in older adults with hypertension, and the results of the meta-analysis showed that the correlation between self-rated health and the occurrence of depression in older adults with hypertension was not significant [OR 95% CI 0.88 (0.58, 1.34), *P* = 0.56]. Four studies (23, 30, 32, 35) analyzed the association between living alone and the occurrence of depression in older adults with hypertension, and statistical heterogeneity (I^2^ = 57%, *P* = 0.07) was found across studies. Sensitivity analysis was conducted and revealed that the study by Jin 2017 (30) was a source of heterogeneity. After excluding this study, the pooled analyses were performed again, and the results showed that living alone was a risk factor for depression in older adults with hypertension [OR 95% CI 1.79 (1.57, 2.04), *P* < 0.001]. Three studies (26, 34, 40) analyzed the correlation between stressful events and the occurrence of depression in older adults with hypertension, and statistical heterogeneity was found across studies (I^2^ = 53%, *P* = 0.12). Sensitivity analysis was conducted and revealed that the study by Ma 2015 (40) was a source of heterogeneity. After excluding this study, the pooled analyses were performed again, and the results showed that stressful events were a risk factor for depression in older adults with hypertension [OR 95% CI 1.62 (1.39, 1.90), *P* < 0.001]. These findings were shown in Figure 5.

## 4 Discussion

### 4.1 Description of evidence

In this study, the occurrence and risk factors for depression in older adults with hypertension in different regions were integrated for the first time by meta-analysis, and the results showed that the incidence of depression in older adults with hypertension was 29%. Female sex, low education level, rural areas, comorbidities, high hypertension classification, non-marital status, poor sleep quality, activity limitation, drinking, low social support, living alone, stressful events, and a long course of disease were risk factors for depression in this population. These findings provide guidance for future health care providers to screen and treat older adults with hypertension who are at risk for depression.

### 4.2 Summary of evidence

The present study showed that the incidence of depression in older adults with hypertension was 29%, which is basically consistent with the results of previous studies (41). The results of the subgroup analysis showed that the incidence of depression in older adults with hypertension in China was 35%, which was higher than the incidence of depression in older adults with hypertension in other countries (16%). This difference may be related to the variation in the included sample sizes. The results of the subgroup analysis showed that the incidence of depression in older adults with hypertension in the cross-sectional study was 28%, and the incidence of depression in older adults with hypertension in the case–control study was 34%. The analysis and comparison revealed that the older adults with hypertension included in the 2 case–control studies (23, 27) were older (≥70), and the studies showed (42) that the functions of various organs, such as the pro-adrenal corticosteroid system, gradually decline with age, increasing the risk of depression in older adults with hypertension. The results of the subgroup analyses showed significant differences in the detection rates of depression in older adults with hypertension among different assessment tools, which may be due to the different areas of focus of different assessment tools. Therefore, health care providers may have the flexibility to utilize one or more assessment tools to comprehensively screen for depression in older adults with hypertension.

The results of this study showed that female sex is a risk factor for depression in older adults with hypertension, which may be due to lower serotonin levels in females compared to males (43), thereby increasing the risk of depression in women. Low level of education and rural residence are risk factors for depression in older adults with hypertension. When patients have a low level of education and live in rural areas, they have limited knowledge about the disease and limited access to professional help, are easily misled by external information, are unable to correctly recognize the disease and are prone to fear the disease, thereby increasing the risk of depression in patients (26, 44). Being unmarried and living alone are also risk factors for depression in older adults with hypertension. Unmarried older adults, especially those living alone, lack caretakers in daily life, lack listeners for emotional expression and are prone to self-doubt, depression, and other negative emotions when facing the disease alone. High hypertension classification, comorbidities, and a long course of disease are risk factors for depression in older adults with hypertension, probably because the higher the hypertension classification, the more severe the disease, the higher the risk of complications, and the longer the disease duration, leading to a lack of confidence in disease treatment and an increased risk of depression among patients (23, 28). Activity limitation and poor sleep quality are risk factors for depression in older adults with hypertension, which is consistent with You et al.'s study (45, 46). The reason for this may be that some older adults with hypertension cannot participate in normal physical activity, thereby inhibiting the secretion of norepinephrine and dopamine hormones. Additionally, poor sleep quality among older adults with hypertension leads to the activation of the hypothalamic pituitary adrenergic system, the functional alteration of the 5-hydroxytryptamine (5-HT) system, and an increase in the production of inflammatory cytokines, which are the potential mechanisms for inducing the development of depression (45, 46). The lower the degree of social support is, the higher the risk of depression in older adults with hypertension (29), which may be because as the function of each organ declines significantly in older adults, their emotional needs are more intense, and they need more support from the outside world. Therefore, it is important to encourage patients' families, friends, and social workers to give them more care and support to help them effectively resolve their negative emotions and reduce the incidence of depression in older adults with hypertension. Drinking is a risk factor for depression in older adults with hypertension, which may be related to the fact that drinking can aggravate the patient's condition. The more severe the patient's condition is, the greater the psychological pressure on the patient, which leads to depression and other negative emotions (28). Stressful events are also risk factors for depression in older adults with hypertension, which may be because in the face of pressure, the body will secrete a hormone called adrenaline, making the body more alert and excited; however, when this state persists, the body will accelerate the heart rate, increase blood pressure, and increase the risk of depression (34, 47).

### 4.3 Highlights and limitations

The study has many highlights. First, this study systematically analyzed the current status of depression occurrence and its risk factors in older adults with hypertension and conducted subgroup analysis and sensitivity analysis to reduce the heterogeneity of studies with large heterogeneity. Second, this study was conducted in strict accordance with the PRISMA guidelines, and the GRADE system was used to rate the quality of evidence, which made this study more rigorous and standardized. Finally, this study was guided by a clinical problem-oriented and evidence-based approach to investigate the current situation of and risk factors for depression in older adults with hypertension, thus providing a preventive guide to reduce the occurrence of depression in older adults with hypertension worldwide.

There are also some limitations in this study. First, this study did not find a significant correlation between self-rated health and monthly income and depression in older adults with hypertension, which may be related to the different countries included in the study and individual differences in the included study subjects. It is suggested that a more rigorous study protocol should be designed in the future. Second, the conclusions of this study are only applicable to older adults with hypertension; further validation is needed before applying these findings to other populations. In addition, to better explore the relationship between hypertension and depression, it is suggested that future scholars further explore the mechanism of sleep and physical activity in hypertension and depression.

## 5 Conclusion

Overall, this study analyzed the occurrence of depression in older adults with hypertension and summarized 13 risk factors through meta-analysis. Medical and health personnel can carry out early screening and management of older adults with hypertension according to the above risk factors to reduce the occurrence of depression in older adults with hypertension. However, most of the study subjects included in this study were from China, and more high-quality studies on the incidence of depression and risk factors in older adults with hypertension worldwide need to be included in the future to improve the quality of evidence.

## Data availability statement

The original contributions presented in the study are included in the article/Supplementary material, further inquiries can be directed to the corresponding authors.

## Author contributions

QG: Conceptualization, Formal analysis, Writing—original draft, Writing—review & editing, Methodology. RY: Conceptualization, Writing—review & editing, Formal analysis. ZL: Formal Analysis, Methodology, Writing—review & editing. YY: Writing—review & editing, Data curation. YL: Writing—review & editing, Data curation. LZ: Funding acquisition, Supervision, Validation, Writing—review & editing.
