# Supplementary material for: Unraveling the link between hypertension and depression in older adults: a meta-analysis
Source: Front Public Health. 2023 Nov 24;11:1302341. doi: 10.3389/fpubh.2023.1302341 (PMC10704466; doi:10.3389/fpubh.2023.1302341)
Supplement: Supplementary file 1 [file Table_1.DOCX]

**Search Strategies**

**1 PubMed（957）**

#1 "Hypertension"[MeSH Terms]

#2 "blood pressure high"[Title/Abstract] OR "blood pressures high"[Title/Abstract] OR "high blood pressure"[Title/Abstract] OR "high blood pressures"[Title/Abstract]

#3 #1 OR #2

#4 "Depression"[MeSH Terms]

#5 "depressive symptoms"[Title/Abstract] OR "depressive symptom"[Title/Abstract] OR "symptom depressive"[Title/Abstract] OR "emotional depression"[Title/Abstract] OR "depression emotional"[Title/Abstract] OR "depressed"[Title/Abstract]

#6 #4 OR #5

#7 "Risk Factors"[MeSH Terms]

#8 "factor risk"[Title/Abstract] OR "risk factor"[Title/Abstract] OR "social risk factors"[Title/Abstract] OR (("Factor"[All Fields] OR "factor s"[All Fields] OR "Factors"[All Fields]) AND "social risk"[Title/Abstract]) OR "factors social risk"[Title/Abstract] OR "risk factor social"[Title/Abstract] OR "risk factors social"[Title/Abstract] OR "social risk factor"[Title/Abstract] OR "health correlates"[Title/Abstract] OR "correlates health"[Title/Abstract] OR "population at risk"[Title/Abstract] OR "populations at risk"[Title/Abstract] OR "risk scores"[Title/Abstract] OR "risk score"[Title/Abstract] OR "score risk"[Title/Abstract] OR "risk factor scores"[Title/Abstract] OR "risk factor score"[Title/Abstract] OR "score risk factor"[Title/Abstract]

#9 "related factor*"[Title/Abstract] OR "influence factor*"[Title/Abstract] OR "factor*"[Title/Abstract]

#10 #7 OR #8 OR #9

#11 #3 AND #6 AND #10

**2 Web of Science (1200)**

#1 (TS=(Elderly hypertension)) OR TS=(hypertension in Elderly)

#2 ((((((TS=(Depression)) OR TS=(depressive symptoms)) OR TS=(depressive symptom)) OR TS=(symptom depressive )) OR TS=(emotional depression)) OR TS=( depression emotional)) OR TS=(depressed )

#3 ((((TS=(Risk Factors)) OR TS=(related factor*)) OR TS=(influence factor*)) OR TS=(factor*)) OR TS=(Risk Factor)

#4 #1 AND #2 AND #3

**3 Embase（5296）**

#1 'elderly hypertension' OR (('elderly'/exp OR elderly) AND ('hypertension'/exp OR hypertension)) OR 'hypertension in elderly':ab,ti

#2 'depression'/exp

#3 'depressive symptoms'/exp OR 'depressive symptoms' OR (depressive AND ('symptoms'/exp OR symptoms)) OR depression:ab,ti OR 'depressive symptom':ab,ti OR 'symptom depressive':ab,ti OR 'emotional depression':ab,ti OR 'depression emotional':ab,ti OR depressed:ab,ti

#4 #2 OR #3

#5 'risk factor'/exp

#6 related AND factor* OR 'influence factor*':ab,ti OR factor*:ab,ti OR 'risk factor':ab,ti

#7 #5 OR #6

#8 #1 AND #4 AND #7

**4 Cochrane (98)**

#1 (Elderly hypertension):ti,ab,kw OR (hypertension in Elderly):ti,ab,kw

#2 MeSH descriptor: [Depression] explode all trees

#3(Depression):ti,ab,kw OR (depressive symptom*):ti,ab,kw OR (emotional depression):ti,ab,kw OR (symptom depressive):ti,ab,kw OR (depressed):ti,ab,kw

#4 #2 OR #3

#5 MeSH descriptor: [Risk Factors] explode all trees

#6(Risk Factor*):ti,ab,kw OR (related factor*):ti,ab,kw OR (influence factor*):ti,ab,kw OR (factor*):ti,ab,kw

#7 #5 OR #6

#8 #1 AND #4 AND #7

**5 知网（CNKI 144）**

#1老年高血压 + 中老年高血压

#2 抑郁 + 抑郁症 + 抑郁情绪 + 负面情绪

#3 影响因素 + 危险因素 + 因素 + 相关因素 + 预测因素

#4 #1 AND #2 AND #3

**6 万方（Wanfang 767）**

#1 老年高血压 OR 中老年高血压

#2 抑郁 OR 抑郁症 OR 抑郁情绪 OR 负面情绪

#3 影响因素 OR 危险因素 OR 因素 OR 相关因素 OR 预测因素

#4 #1 AND #2 AND #3

**7 维普（**VIP **38）**

#1 老年高血压 OR 中老年高血压

#2 抑郁 OR 抑郁症 OR 抑郁情绪 OR 负面情绪

#3 影响因素 OR 危险因素 OR 因素 OR 相关因素 OR 预测因素

#4 #1 AND #2 AND #3

**8 SinoMed(114)**

#1 "老年高血压"[常用字段:智能] OR "中老年高血压"[常用字段:智能]

#2 "抑郁"[不加权:扩展]

#3 "抑郁"[常用字段:智能] OR "抑郁症"[常用字段:智能] OR "抑郁情绪"[常用字段:智能] OR "负面情绪"[常用字段:智能]

#4 (#2) OR (#3)

#5 "危险因素"[不加权:扩展]

#6 "影响因素"[常用字段:智能] OR "危险因素"[常用字段:智能] OR "因素"[常用字段:智能] OR "相关因素"[常用字段:智能] OR "预测因素"[常用字段:智能]

#7 (#5) OR (#6)

#8 (#1) AND (#4) AND (#7)
